# Intraoperative neurological pupil index and postoperative delirium and neurologic adverse events after cardiac surgery: an observational study

**DOI:** 10.1038/s41598-023-41151-z

**Published:** 2023-08-24

**Authors:** Seohee Lee, Dhong-Eun Jung, Dongnyeok Park, Tae Jung Kim, Hyung-Chul Lee, Jinyoung Bae, Karam Nam, Yunseok Jeon, Youn Joung Cho

**Affiliations:** 1 Department of Anesthesiology and Pain Medicine, Seoul National University Hospital, Seoul National University College of Medicine, 101 Daehak-Ro, Jongno-Gu, Seoul, 03080 Republic of Korea; 2 Dasom Anesthesia and Analgesia Practice Association, Seoul, Republic of Korea; 3 Department of Critical Care Medicine, Seoul National University Hospital, Seoul National University College of Medicine, Seoul, Republic of Korea

**Keywords:** Medical research, Neurology, Risk factors

## Abstract

Neurological pupil index (NPi) calculated by automated pupillometry predicts clinical outcomes in critically ill patients. However, there are few data on intraoperative NPi and postoperative outcome after cardiac surgery. We evaluated the relationships between intraoperative NPi and clinical outcomes, such as delirium, in cardiac surgery patients. NPi was measured at baseline, after anesthesia induction, at 30 min intervals after initiation of cardiopulmonary bypass or anastomosis of coronary artery bypass graft, and at skin closure. Abnormal NPi was defined as one or more measurements of NPi < 3.0 during surgery. The worst intraoperative NPi was recorded, then multivariate logistic regression analysis was performed to evaluate the relationship between abnormal NPi and postoperative delirium following cardiac surgery. Among 123 included patients, postoperative delirium developed in 19.5% (24/123) of patients. Intraoperative abnormal NPi was significantly associated with postoperative delirium (odds ratio 6.078; 95% confidence interval 1.845–20.025; P = 0.003) after adjustment for Society of Thoracic Surgeons Predicted Risk of Mortality score, coronary artery disease, and use of calcium channel blockers. In conclusion, abnormal intraoperative NPi independently predicted postoperative delirium following cardiac surgery. Intraoperative application of pupillometry may have prognostic value for development of postoperative delirium, thereby enabling close surveillance and early intervention in high-risk patients.

Registry number: ClinicalTrials.gov (NCT04136210).

## Introduction

Postoperative delirium is a common complication after major surgery especially following cardiac surgery^1^. Its incidence varies among literature, ranging up to 55% after cardiac surgery^2^. Postoperative delirium increases morbidity (prolonged hospital stay, institutionalization, and persistent functional disturbance), perioperative mortality, and health care burden^1,3^. However, there are currently no available intraoperative tools to predict or monitor postoperative delirium after surgery.

Automated digital pupillometry is an emerging modality for objective evaluation of pupillary light reflex and provides quantitative parameters in response to standardized light stimulus^4^. The neurological pupil index (NPi) is calculated based on parameters obtained from automated pupillometric devices, and an abnormal NPi predicts poor clinical outcome in patients after cardiac arrest^5^. In a cross-sectional cohort study, post-illumination pupillary reflex using infrared pupillometer showed a modest correlation with cognitive function scores, suggesting the role of pupillary response in predicting cognitive outcome in middle- and old-aged adults^6^. An observational study of medico-surgical critical patients revealed that sustained abnormalities of quantitative pupillary reactivity were associated with an increased risk of delirium, suggesting the pathophysiological concept of the relationship of a particular neurocritical illness and deterioration of brainstem reflexes, which can be clinically detected^7,8^. Pupillary light reflexes can be assessed during surgery and under extracorporeal bypass, thus can be a robust predictor of postoperative delirium, which is an important complication after cardiac surgery.

However, few studies have evaluated the association between intraoperative NPi and postoperative clinical outcomes, including delirium, in patients undergoing cardiac surgery. We hypothesized that intraoperative measures of objective pupillometry would have a predictable value for postoperative clinical outcomes, such as delirium, in patients undergoing cardiac surgery. To evaluate our hypothesis, we assessed the relationships of intraoperative NPi using automated digital pupillometry and postoperative delirium after cardiac surgery.

## Methods

### Study design and ethical approval

This was a prospective observational study conducted as a sub-analysis of the Registry of Perioperative Data in Cardiovascular Surgery at Seoul National University Hospital, which was approved by the Institutional Review Board of Seoul National University Hospital (#1908–095–1055; principal investigator, Yunseok Jeon; on September 9, 2019) and registered at ClinicalTrials.gov (NCT04136210; on October 23, 2019). The purpose of the registry was to obtain perioperative data in patients undergoing cardiovascular surgery and to investigate the relationships of data and postoperative complications. In the registry, we performed automated digital pupillometry to measure ‘pupillary reactivity assessment scale’ and assessed postoperative delirium as ‘neurologic outcomes’, which is described in ClinicalTrials.gov. All methods were performed in accordance with the relevant guideline and regulations, including Strengthening the Reporting of Observational Studies in Epidemiology (STROBE) guidelines^9^. Written informed consent was obtained from all participants, who could withdraw at any time.

### Patient population

This study included adult patients (age ≥ 20 years) who were undergoing elective cardiac surgery, including coronary artery bypass graft, cardiac valve surgery, ascending aorta or aortic arch surgery, or a combination thereof, and who performed intraoperative pupillometric assessment during surgery. Patients who were undergoing descending thoracic aorta surgery, preoperatively comatose, with primary acute or known neurological deficit, or intentionally sedated postoperatively were excluded. The registry initially recruited adult patients undergoing cardiac or thoracic aorta surgery (NCT04136210). We screened all patients enrolled in the registry, and eligible patients were included in this sub-study to assess pupillometry during the study period.

### Anesthetic management and cardiopulmonary bypass strategy

Patients were monitored with five-lead electrocardiography, invasive blood pressure, pulse oxygen saturation, bispectral index (BIS), and cerebral oxygen saturation using non-infrared spectroscopy (INVOS 5100C Oximeter, Covidien, Mansfield, MA, USA). General anesthesia was induced with administration of midazolam 0.1 mg/kg and sufentanil 1 μg/kg intravenously. Anesthesia was maintained using target-controlled infusions of propofol (effect site concentration [Ce] 1–2 μg/ml) and remifentanil (Ce 3–5 ng/ml).

Coronary artery bypass graft was performed using an on- or off-pump strategy as per the surgeon’s discretion. Cardiopulmonary bypass (CPB) was performed using a circuit incorporating a membrane oxygenator primed with Ringer’s lactated solution 1–1.5 l, 15% mannitol 300 ml, 20% albumin 100 ml, sodium bicarbonate, and heparin. Systemic heparinization was established by administering intravenous heparin 3 mg/kg before cannulation. Left ventricular (LV) venting was performed through the right superior pulmonary vein. Non-pulsatile bypass was performed (Advanced Perfusion System 1, Terumo Cardiovascular Systems Corporation, Ann Arbor, MI, USA) with a pump flow rate of 2–2.5 l/min/m^2^ of the body surface area; α-stat pH management was implemented under mild (28–32 °C) or moderate (23–28 °C) hypothermia. During bypass, the mean arterial pressure was maintained at 50–80 mmHg, hematocrit 20–25%, and activated clotting time > 400 s. Intraoperative cell salvage was used, and myocardial protection was supported by cold cardioplegic arrest. Brain protection was monitored using a cerebral oximeter. After weaning from the CPB, heparinization was reversed by administration of protamine. Patients were transferred to the cardiopulmonary intensive care unit (ICU), and were extubated once adequate spontaneous breathing and conscious level were achieved. During ICU stay, dexmedetomidine was used in patients presenting severe agitation and those weaning from mechanical ventilation. Use of benzodiazepine was minimized, and postoperative pain was adequately assessed and controlled, as a part of our institutional standardized protocol to reduce delirium.

### Quantitative pupillometric assessment

The pupillary light reflex was assessed using a portable automated NPi-200 pupillometer (NeurOptics, Laguna Hills, CA, USA), which uses an infrared camera that integrates fixed light stimulation at an intensity of 1000 lx and duration of 3.2 s. The device analyses the pupillary response to a standard light stimulus, including basal and minimum diameters, percent change in pupil diameter, dilatation velocity, pupil constriction velocity, maximal constriction velocity and latency of constriction (expressed as time onset of constriction following initiation of the light stimulus). NPi was calculated based on the responsive variables and integrated algorithm and expressed as a unitless score between 0 and 5 (with a 0.1 decimal precision). As the integrated NPi value is known to be minimally affected by sedatives or analgesics compared to individual responsive parameters, we used the NPi value for analysis in the study^10,11^. NPi ≥ 3.0 was considered a normal reflex, and < 3.0 was considered abnormal reflex^12^. Complete absence of a pupillary reflex correlated with an NPi value of zero. Pupillometry assessment was performed at baseline, after anesthesia induction, at initiation of CPB or coronary artery bypass grafting, at 30 min intervals thereafter, at protamine infusion, and at the end of surgery. When there were discrepancies between the NPi value for the right and left pupils, the worse NPi was included in the analysis. Abnormal intraoperative NPi was defined as one or more measurements of NPi < 3.0 during the surgery.

### Primary and secondary endpoints

The primary endpoint was the relationship between the abnormal intraoperative NPi during the surgery and development of postoperative delirium. For secondary endpoints, we compared the incidences of other clinical outcomes, including stroke, acute kidney injury, newly initiated continuous renal replacement therapy, requirement for mechanical circulatory support device (extracorporeal membrane oxygenation, intra-arterial balloon pump, or ventricular assist device), and in-hospital mortality, between patients with the worst intraoperative NPi ≥ 3.0 and < 3.0 during the surgery.

Delirium was evaluated using the 3-min Diagnostic Confusion Assessment Method (3D-CAM) or Confusion Assessment Method for the Intensive Care Unit (CAM-ICU)^13^. 3D-CAM and CAM-ICU evaluates four features of delirium: acute onset, inattention, altered level of consciousness, and disorganized thinking^14,15^. Delirium was assessed at least three times per day, including 8 A.M. in the morning, and whenever patient’s altered consciousness was suspected, until 7 days postoperatively. Presence of delirium was not evaluated when the patients were intentionally sedated and was excluded from analysis.

Stroke was defined as an acute episode of focal or global neurologic deficit lasting for ≥ 24 h, with at least one of the following: altered level of consciousness, hemiplegia, hemiparesis, numbness or sensory loss affecting one side of the body, dysphagia or aphagia, hemianopia, amaurosis fugax, or other neurologic signs or symptoms consistent with stroke, which was finally confirmed by neuroimaging studies, such as computed tomography or magnetic resonance imaging. Postoperative AKI was determined on the basis of serum creatinine level and urine output according to the criteria of the Kidney Disease: Improving Global Outcomes Clinical Practice Guidelines for Acute Kidney Injury^16^. AKI was defined as ≥ 1.5-fold increase in serum creatinine from the baseline level, increase of ≥ 0.3 mg/dl, or urine output < 0.5 ml/kg/h for ≥ 6 h within 7 days of surgery.

### Other data collection

The baseline characteristics of patients, perioperative hemodynamic and intraoperative variables were collected. Intraoperative moderate (≥ 25% reduction from baseline) or severe (≥ 50% reduction from baseline) cerebral desaturation was recorded, using the criteria from previous studies to evaluate the severity of cerebral desaturation based on the initial values^17,18^.

### Statistical analyses

Data are presented as mean (standard deviation), median (interquartile range [IQR]), or number (proportions). The normality of data was tested using the Kolmogorov–Smirnov test. Continuous variables were compared using the independent *t*-test or Mann–Whitney U test. Categorical variables were compared using the Pearson’s chi-square test or Fisher’s exact test. The repeatedly measured data were analyzed using linear mixed models. In the mixed model, the event, time, and interaction between event and time were considered fixed effects, whereas subject was considered a random effect. When the interaction between the event and time was significant, the mean difference in NPi between the event groups at each measurement time was estimated by using a linear contrast in the linear mixed model and the P value from the linear contrast test was multiplied by 6 for Bonferroni correction for the multiple comparison since the group comparison was made at 6 measurement time points (after anesthesia induction, at initiation of CPB or coronary artery bypass, at 30 min, 60 min of CPB or coronary artery bypass, at protamine infusion, and at the end of surgery). For repeatedly measured variables, residuals versus fitted value plots were used to confirm that the error terms (residuals) had a mean of zero and constant variance. The normality of data was assessed using histograms and normal quantile–quantile plots of residuals.

To identify the independent risk of intraoperative NPi for postoperative delirium, a multivariable logistic regression model was used, which included the potential covariates known to affect postoperative outcome after cardiac surgery. For multivariable regression analysis, we considered patient baseline characteristics, comorbidity, preoperative medications, and intraoperative variables to estimate odds ratios (ORs) for postoperative delirium. Because of the small number of data, variables to be included in the multivariable logistic regression model were selected using stepwise selection among the variables with P < 0.2 in univariable analyses. Univariable logistic regression analysis for type of surgery was performed using Firth penalized maximum likelihood because logistic regression failed to converge due to no delirium occurrence in those underwent aorta surgery (Table 1). We performed logistic regression analysis using the NPi value as both continuous and categorical variables, which was dichotomized with the cut-off limit of 3.0 according to the manufacturer’s instructions and previous studies^5,10^, in a separate analysis. We also assessed the risks of intraoperative abnormal NPi for postoperative delirium in two additional sub-groups of patients who used CPB during the surgery and those who underwent cardiac valve surgery as sub-analyses to reduce the impact of heterogeneity of included surgery.

**Table 1:** Baseline characteristics of patients with and without postoperative delirium after cardiac surgery.

	No postoperative delirium (n = 99)	Postoperative delirium (n = 24)	P value
Demographic data			
Age, years	63 (range, 29–87)	72 (range 48–81)	0.009
Male	61 (61.6%)	13 (54.2%)	0.504
Height, cm	161.9 (9.5)	157.9 (8.3)	0.067
Weight, kg	63.8 (13.3)	60.0 (11.3)	0.199
Body mass index, kg/m^2^	24.2 (3.6)	23.9 (3.3)	0.718
Body surface area, m^2^	1.68 (0.24)	1.62 (0.19)	0.264
STS-PROM, %	1.21 (0.66–2.39)	2.51 (1.62–6.14)	< 0.001
Preoperative LV EF, %	60 (55–63)	57 (46–63)	0.166
Baseline hematocrit, %	39.2 (5.2)	36.2 (6.9)	0.020
Baseline hs-CRP, mg/dl	0.09 (0.04–0.23)	0.21 (0.06–0.41)	0.058
Smoking status			0.769
Never smoker	76 (76.8%)	20 (83.3%)	
Current smoker	16 (16.2%)	3 (12.5%)	
Ex-smoker	7 (7.1%)	1 (4.2%)	
Comorbidity			
Hypertension	49 (49.5%)	13 (54.2%)	0.681
Diabetes	33 (33.3%)	10 (41.7%)	0.442
Coronary artery disease	34 (34.3%)	13 (54.2%)	0.073
Previous MI or angina	23 (23.2%)	7 (29.2%)	0.544
Chronic kidney disease	12 (12.1%)	8 (33.3%)	0.012
Preoperative atrial fibrillation	29 (29.3%)	14 (58.3%)	0.007
Previous stroke or TIA	8 (8.1%)	4 (16.7%)	0.203
COPD	6 (6.1%)	3 (12.5%)	0.277
Preoperative medication			
ACEi or ARB	47 (47.5%)	9 (37.5%)	0.379
Beta blocker	55 (55.6%)	12 (50.0%)	0.624
Calcium channel blocker	51 (51.5%)	8 (33.3%)	0.110
Diuretics	44 (44.4%)	16 (66.7%)	0.051
Statin	62 (62.6%)	13 (54.2%)	0.446
Benzodiazepine	5 (5.1%)	4 (16.7%)	0.072
Type of surgery			0.696
CABG	26 (26.3%)	8 (33.3%)	
Valve surgery	47 (47.5%)	12 (50.0%)	
Aorta surgery	7 (7.1%)	0 (0%)	
Aortic arch surgery	4 (4.0%)	0 (0%)	
Combined surgery*	11 (11.1%)	2 (8.3%)	
Other cardiac surgery^†^	8 (8.1%)	2 (8.3%)	
Redo surgery	8 (8.1%)	5 (20.8%)	0.130
Postoperative ICU stay, h	49 (27–93)	98 (57–180)	< 0.001
Postoperative hospital stay, day	14 (12–20)	24 (19–34)	< 0.001

Values are mean (standard deviation), number (proportions), or median (interquartile range). Age was presented as median (range).

*ACEi* angiotensin converting enzyme inhibitor, *ARB* angiotensin II receptor blocker, *CABG* coronary artery bypass graft, *COPD* chronic obstructive pulmonary disease, *EF* ejection fraction, *hs-CRP* high sensitivity C-reactive protein, *ICU* intensive care unit, *LV* left ventricle, *MI* myocardial infarction, *STS-PROM* the Society of Thoracic Surgeons Predicted Risk of Mortality, *TIA* transient ischemic attack.

*Combined surgery included concomitant valve, aorta, and/or coronary artery bypass graft surgery.

^†^Other cardiac surgery included repair of atrial septal defect, excision of intracardiac mass, myectomy, and endoventricular circular patch plasty.

Pearson’s correlation analyses were used to evaluate the relationships between the worst intraoperative NPi and variables known to affect consciousness, such as lowest intraoperative BIS, minimum cerebral oxygen saturation, minimum core body temperature, and remifentanil administration.

Additionally, the best cut-off limit of intraoperative worst NPi associated with postoperative delirium was identified using receiver operating characteristic (ROC) curve analysis. The cut-off with maximum sensitivity and specificity was estimated using the Youden J index. The area under the ROC curve (AUC) and the corresponding 95% CI were calculated.

We recruited eligible patients during the study period without sample size calculation for this observational study. Then we calculated the estimated power of the study after data analysis based on a previous study, in which the association of NPi and delayed cerebral ischemia (defined as a new focal neurological deficit or sudden deterioration in the level of consciousness) was evaluated^19^.

Statistical analyses were performed using SPSS (version 21.0; IBM Corp., Armonk, NY, USA), MedCalc® (version 20.019, MedCalc Software Ltd., Ostend, Belgium), SAS (version 9.4. SAS Institute Inc., Cary, NC, USA), and R software (version 3.4.3, R Foundation for Statistical Computing, Vienna, Austria) for Microsoft Windows. A post-hoc power was computed using PASS 2022 (NCSS, LLC., Kaysville, UT, USA). A P value < 0.05 was considered statistically significant.

## Results

Among 136 patients undergoing cardiovascular surgery between September 19 and December 24, 2019, 133 patients were registered in the registry except for 3 who refused to participate. We excluded 10 patients who undergoing concomitant noncardiac surgery (n = 1), whose operation schedule was delayed (n = 6), undergoing descending thoracic aorta surgery (n = 1), had preoperative neurological deficit (n = 1), and who received ophthalmic surgery (n = 1), then the remaining 123 patients were eligible and were included in this study to perform intraoperative pupillometric assessment during the cardiac surgery (see Supplementary Fig. S1 online). The median age was 65 (IQR 58–73, range 29–87) years, and 61.6% of patients were males. Baseline patient characteristics are presented in Table 1.

Postoperative delirium developed in 24/123 (19.5%) of patients during hospitalization, diagnosed at a median 2 (1–4) days postoperatively. Postoperative delirium occurred more frequently in patients who had intraoperative worst NPi < 3.0 (35.5%, 11/31) compared with those maintained NPi ≥ 3.0 throughout the surgery (17.4%, 16/92). Other incidences of postoperative clinical outcomes were comparable between patients with intraoperative worst NPi ≥ 3.0 and < 3.0 (see Supplementary Table S1 online).

Perioperative variables are presented in Table 2. Depth of anesthesia, assessed by BIS, was comparable between the groups throughout the surgery. Cerebral oxygen saturations were lower in patients with postoperative delirium during surgery, while there was no difference at the end of surgery (Table 2). The duration of surgery was longer and intraoperative transfusion was more frequent in patients developed postoperative delirium. Dexmedetomidine was used in all patients until weaning from mechanical ventilation postoperatively during ICU stay, and no patient received dexmedetomidine thereafter. Postoperative use and total dose of benzodiazepines were higher in patients with delirium compared to those without delirium (Table 2).

**Table 2:** Perioperative variables in patients with and without postoperative delirium after cardiac surgery.

	No postoperative delirium (n = 99)	Postoperative delirium (n = 24)	P value
Worst intraoperative NPi of 0	17 (17.2%)	7 (29.2%)	0.183
Bispectral index			
Baseline	93 (11)	95 (4)	0.472
At initiation of CPB or coronary artery bypass	37 (11)	39 (9)	0.526
At protamine infusion	44 (10)	43 (10)	0.825
End of surgery	44 (8)	45 (6)	0.837
Lowest bispectral index	29 (14)	28 (11)	0.692
Cerebral oximeter, %			
Right			
Baseline	62 (10)	57 (9)	0.009
At initiation of CPB or coronary artery bypass	62 (11)	50 (13)	< 0.001
At protamine infusion	66 (11)	56 (17)	0.012
End of surgery	63 (8)	58 (12)	0.068
Left			
Baseline	62 (9)	57 (8)	0.007
At initiation of CPB or coronary artery bypass	63 (10)	53 (13)	< 0.001
At protamine infusion	66 (11)	59 (13)	0.010
End of surgery	62 (8)	59 (11)	0.112
Incidence of cerebral desaturation			
Moderate (< 25% of baseline)			
Right*	16 (16.3%)	8 (33.3%)	0.084
Left	16 (16.2%)	5 (20.8%)	0.557
Severe (< 50% of baseline)			
Right*	2 (1.2%)	2 (8.3%)	0.173
Left	2 (2.0%)	1 (4.2%)	0.482
Duration of operation, min	293 (87)	339 (81)	0.018
Use of CPB	75 (75.8%)	18 (75.0%)	0.938
Lowest core body temperature, °C	29.8 (28.0–34.0)	30.0 (27.5–34.4)	0.536
Intraoperative transfusion of any blood product	51 (51.5%)	18 (75.0%)	0.038
Total amount of administered remifentanil, µg	3061 (1550)	2980 (1066)	0.809
Intraoperative use of inotropic or vasoactive agent			
Epinephrine	9 (9.1%)	4 (16.7%)	0.717
Norepinephrine	77 (77.8%)	20 (83.3%)	0.550
Dobutamine	66 (66.7%)	18 (75.0%)	0.431
Nitroglycerin	52 (52.5%)	15 (62.5%)	0.379
Peak hs-CRP within 7 days postoperatively	16.03 (9.80–21.92)	16.63 (10.86–24.08)	0.515
Postoperative use of benzodiazepines	6 (6.1%)	7 (29.2%)	0.001
Total dose of postoperative benzodiazepines, mg	0 (0–0)	0 (0–3)	0.001

Values are mean (standard deviation), number (proportions), or median (interquartile range).

*CPB* cardiopulmonary bypass, *hs-CRP* high sensitivity C-reactive protein, *NPi* neurological pupil index.

*One patient with missing data was excluded.

**Figure 1:**
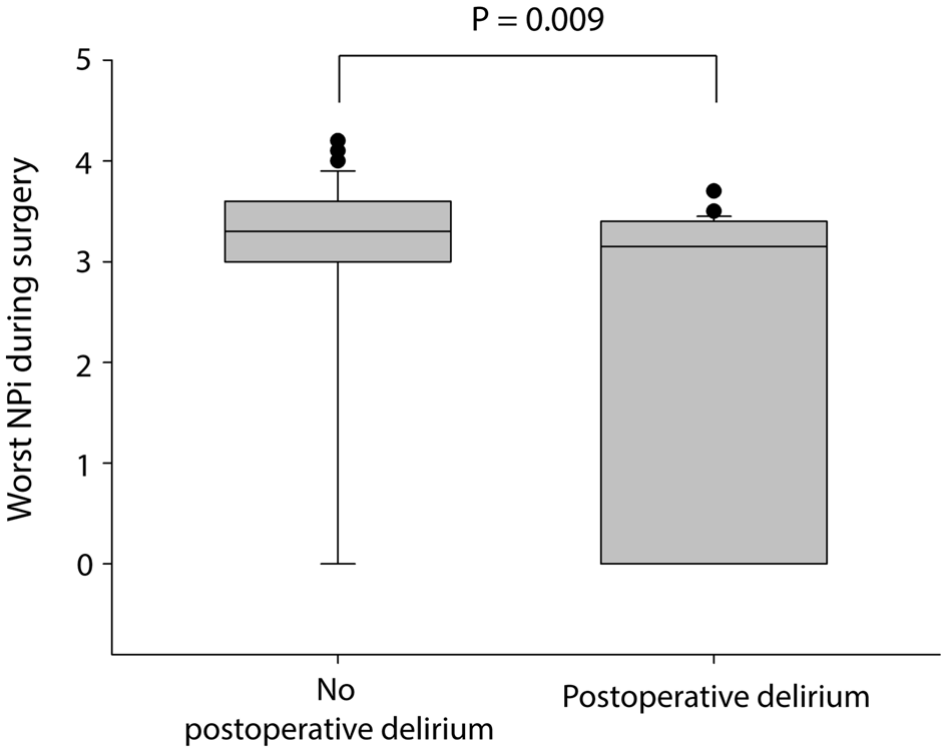
Box plot of the worst neurological pupil index during surgery in patients with and without postoperative delirium. Horizontal line within the box indicates median value. Lower and upper boundaries of the box indicate 25th and 75th percentiles, respectively. Horizontal lines outside the box indicate the minimum and the maximum values of the data. Dots outside the box are outliers. P value between the groups was calculated using Mann–Whitney U test. *NPi* neurological pupil index.

**Figure 2:**
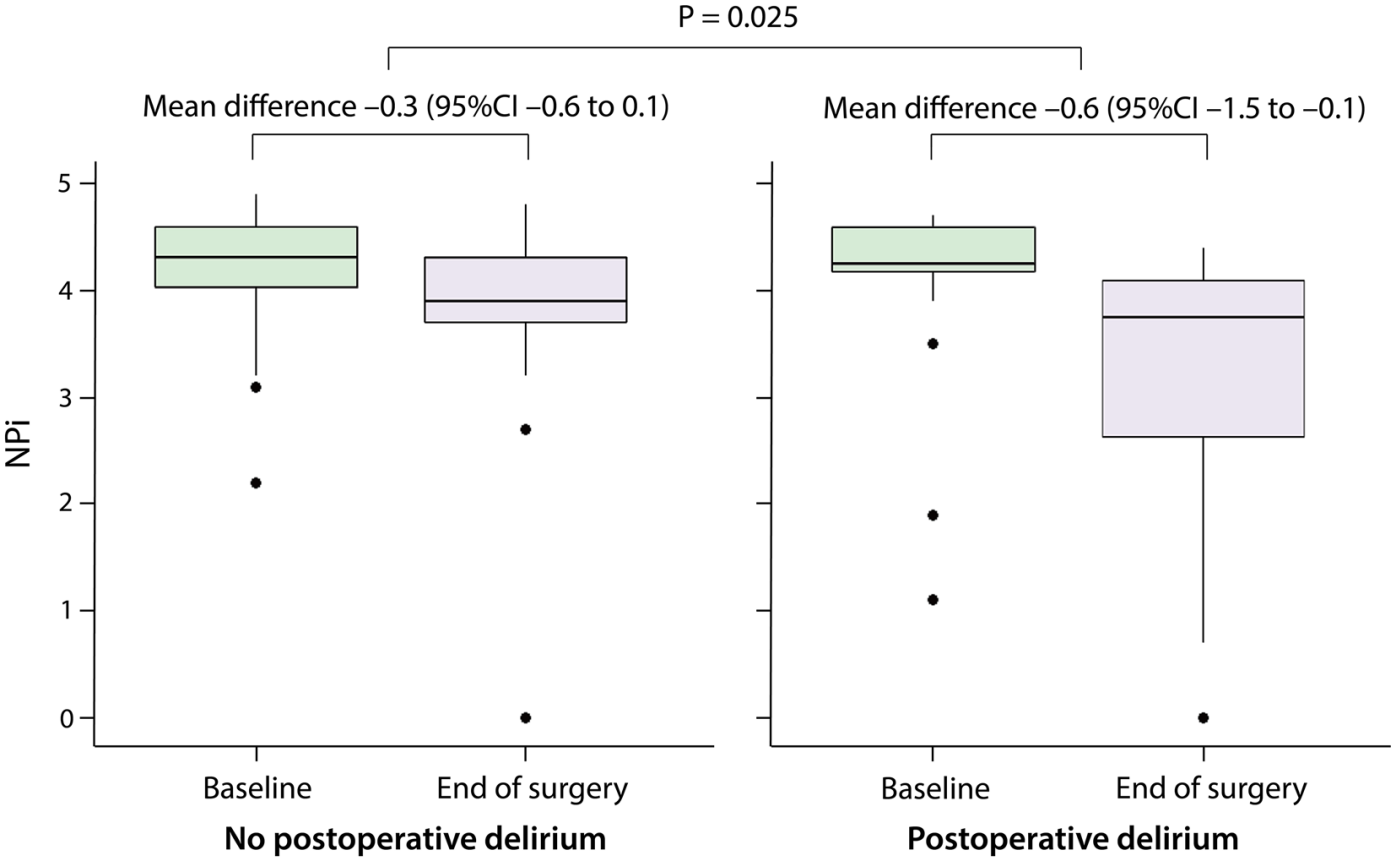
Comparison of neurological pupil index at baseline and at the end of surgery in patients with and without postoperative delirium. *CI* confidence interval, *NPi* neurological pupil index.

Baseline NPi was comparable between patients with and without postoperative delirium (4.3 [4.1–4.6] *vs* 4.3 [4.0–4.6], respectively; P = 0.778). The worst intraoperative NPi was lower in patients who developed postoperative delirium compared to those who did not (median [IQR], 3.2 [0.0–3.4] *vs* 3.3 [3.0–3.6], respectively; P = 0.009; Fig. 1). The sensitivity of intraoperative NPi < 3.0 for postoperative delirium was 41.7% and its specificity was 78.8%. Compared to baseline, NPi decreased greater in patients with postoperative delirium at the end of surgery than those did not (P = 0.025 between the groups, Mann–Whitney *U* test; Fig. 2).

**Figure 3:**
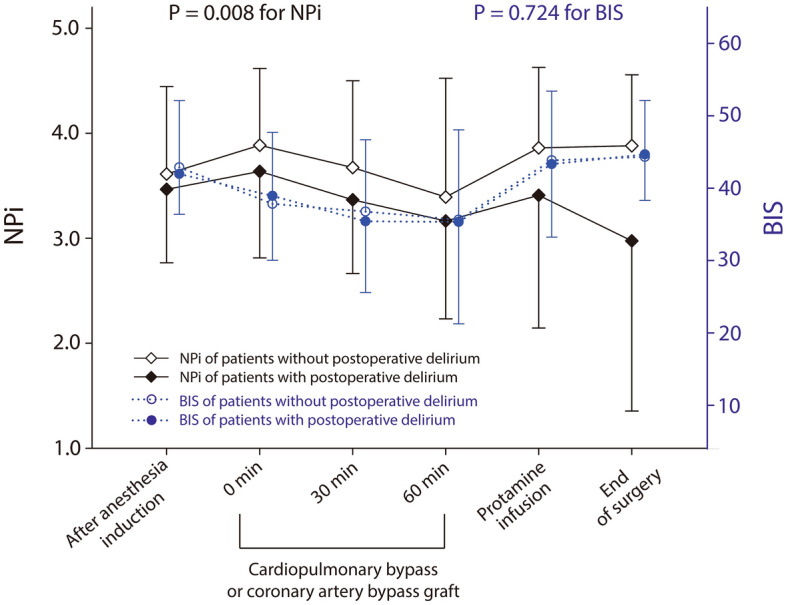
Serial measures of neurological pupil index during surgery in patients with and without postoperative delirium. P value between the groups was calculated using linear mixed model. *NPi* neurological pupil index.

Serial measurements of intraoperative NPi, which were lower in patients with postoperative delirium compared to those without delirium (mean difference − 0.4, 95% CI − 0.7 to − 0.1, P = 0.008, mixed model) are presented in Fig. 3 along with serial BIS values, which showed no difference between the groups (P = 0.724). The interactions between measurement time and event were not significant in the linear mixed model (P = 0.066 and 0.377 for NPi and BIS, respectively).

Among 123 patients, 17/99 (17.2%) and 7/24 (29.2%) patients with and without postoperative delirium, respectively, had worst NPi value of 0 during surgery. Baseline characteristics and perioperative variables of patients with worst intraoperative NPi 0 are presented in Supplementary Tables S2 and S3 online. Intraoperative NPi was significantly lower during CPB or coronary artery bypass and at the end of surgery in patients presented with worst NPi 0 during surgery compared to those did not (see Supplementary Table S3 online). BIS values were significantly lower in patients with worst NPi of 0 compared to those without throughout the surgery (see Supplementary Table S3 online). In scatter plots, the worst intraoperative NPi had a moderate correlation with the lowest BIS (*r* = 0.496, P < 0.001; see Supplementary Fig. S2 online).

After adjustment for covariates, intraoperative abnormal NPi (< 3.0) was significantly associated with postoperative delirium (adjusted OR 6.078; 95% CI 1.845–20.025, P = 0.003; Table 3). When analyzed as a continuous value, maintaining high intraoperative NPi was associated with reduction in risk of postoperative delirium occurrence (adjusted OR 0.621; 95% CI 0.438–0.881, P = 0.008; see Supplementary Table S4 online), which means that each increase of 1 in intraoperative worst NPi was associated with 38% reduction in the odds of postoperative delirium after adjusting covariables. When excluding patients presented with the worst intraoperative NPi of 0, incidence of delirium was 17.2% (17/99) and abnormal NPi (< 3.0) had adjusted OR of 5.997 (95% CI 0.869–41.374; P = 0.069) for postoperative delirium (see Supplementary Table S5 online). Additionally, NPi value of 0 at the end of surgery was associated with increased risk of postoperative delirium (unadjusted OR 9.700; 95% CI 1.662–56.624, P = 0.012).

**Table 3:** Multivariable logistic regression analysis for postoperative delirium in patients undergoing cardiac surgery.

	Unadjusted model		Adjusted model	
	OR (95% CI)	P value	OR (95% CI)	P value
Intraoperative pupillometry				
NPi ≥ 3.0	Ref		Ref	
NPi < 3.0	2.653 (1.032–6.817)	0.043	6.078 (1.845–20.025)	0.003
Baseline characteristics				
Age	1.069 (1.016–1.126)	0.011		
Male	1.358 (0.553–3.339)	0.505		
Body mass index	0.976 (0.859–1.110)	0.716		
Hematocrit	0.908 (0.836–0.987)	0.024		
STS-PROM	1.410 (1.163–1.710)	< 0.001	1.566 (1.218–2.013)	< 0.001
LV EF	0.960 (0.922–1.001)	0.053		
Comorbidity				
Hypertension	1.206 (0.493–2.950)	0.682		
Diabetes mellitus	1.429 (0.573–3.559)	0.444		
Coronary artery disease	2.259 (0.915–5.578)	0.077	6.421 (1.839–22.426)	0.004
Previous MI or angina	1.361 (0.502–3.684)	0.545		
Chronic kidney disease	3.625 (1.279–10.270)	0.015		
Preoperative atrial fibrillation	3.379 (1.347–8.477)	0.009		
Previous stroke or TIA	2.275 (0.624–8.299)	0.213		
Chronic obstructive pulmonary disease	2.214 (0.512–9.578)	0.287		
Preoperative medication				
ACEi or ARB	0.664 (0.266–1.659)	0.381		
Beta blocker	0.800 (0.328–1.954)	0.624		
Calcium channel blocker	0.471 (0.185–1.200)	0.114	0.257 (0.077–0.857)	0.027
Diuretics	2.500 (0.980–6.379)	0.055		
Statin	0.705 (0.287–1.735)	0.447		
Benzodiazepine	3.760 (0.927–15.256)	0.064		
Intraoperative variables				
Duration of operation	1.006 (1.001–1.011)	0.024		
Use of cardiopulmonary bypass	0.960 (0.342–2.694)	0.938		
Type of surgery		0.895		
CABG	Ref			
Valve surgery	0.820 (0.306–2.274)	0.699		
Aorta surgery	0.208 (0.002–2.040)	0.330		
Combined surgery*	0.678 (0.114–2.971)	0.638		
Other cardiac surgery^†^	0.917 (0.151–4.225)	0.919		
Redo surgery	2.993 (0.882–10.158)	0.079		
Lowest core body temperature	1.035 (0.913–1.173)	0.588		
Lowest bispectral index	0.993 (0.961–1.027)	0.689		
Moderate desaturation of cerebral oximeter	1.750 (0.662–4.626)	0.259		
Severe desaturation of cerebral oximeter	3.393 (0.706–16.305)	0.127		
Total amount of infused remifentanil	1.000 (1.000–1.000)	0.807		
Intraoperative transfusion	2.824 (1.034–7.710)	0.043		
Intraoperative use of inotropic or vasoactive agent				
Epinephrine	2.000 (0.560–7.147)	0.286		
Norepinephrine	1.429 (0.442–4.619)	0.551		
Dobutamine	1.500 (0.544–4.135)	0.433		
Nitroglycerin	1.506 (0.603–3.764)	0.381		
Postoperative use of benzodiazepine	6.382 (1.909–21.334)	0.003		

*ACEi* angiotensin converting enzyme inhibitor, *ARB* angiotensin, *CABG* coronary artery bypass graft, *CI* confidence interval, *EF* ejection fraction, *LV* left ventricle, *MI* myocardial infarction, *NPi* neurological pupil index, *OR* odds ratio, *STS-PROM* the Society of Thoracic Surgeons Predicted Risk of Mortality, *TIA* transient ischemic attack.

*Combined surgery included concomitant valve, aorta, and/or coronary artery bypass graft surgery.

^†^Other cardiac surgery included repair of atrial septal defect, excision of intracardiac mass, myectomy, and endoventricular circular patch plasty.

In sub-group analyses, intraoperative abnormal NPi was not associated with postoperative delirium in patients who used CPB during the surgery (n = 93; adjusted OR 2.036; 95% CI 0.590–7.021, P = 0.260; see Supplementary Table S6 online) and in those who underwent cardiac valve surgery (n = 59; adjusted OR 4.357; 95% CI 0.730–25.991, P = 0.106; see Supplementary Table S7 online). The incidences of delirium in the sub-groups were 19.4% (18/93) and 20.3% (12/59), respectively.

ROC curve analysis revealed that the best cut-off limit of intraoperative worst NPi to predict postoperative delirium was 3.4 (AUC 0.670; 95% CI 0.580–0.753, P = 0.002) with sensitivity 91.7% and specificity 38.4%. In multivariable logistic regression analysis with the cut-off limit of 3.4 for intraoperative worst NPi, adjusted OR for postoperative delirium was 2.589 (95% CI 0.784–8.547, P = 0.119).

A post-hoc power calculation indicated that a sample size of 123 patients with 24 postoperative delirium events had 74% power in this study at a significance level of 0.05 to detect an association between NPi and postoperative delirium when the OR for the association was assumed to be 3.393 based on the previous study^19^.

## Discussion

Abnormal NPi during cardiac surgery was significantly associated with postoperative delirium after adjustment for covariates.

The etiology of post-cardiac surgery delirium is complex and multifactorial^20^. Neuroinflammation and altered functional connectivity of the brain with increasing proinflammatory cytokines have been suggested to contribute to postoperative delirium^21^. Risk factors for post-cardiac surgery delirium included age, diabetes mellitus, preoperative depression, cognitive impairment, carotid artery stenosis, electrolyte imbalance, prolonged duration of surgery, and length of mechanical ventilation and ICU stay^22^. Both on- and off-pump cardiac surgeries were major risk factors for postoperative delirium^23^.

Pupil size and reactivity reflect midbrain function^24^. Abnormal pupil light reflex is a well-studied predictor associated with neurologic and clinical adverse outcomes in critically ill patients. In patients resuscitated from cardiac arrest, abnormal NPi (≤ 2.0) at 24 h was a predictor of unfavorable neurological outcome, assessed by cerebral performance at 3 months^25^. An observational cohort of sedated, mechanically ventilated patients on venoarterial extracorporeal membrane oxygenation treatment have demonstrated significantly lower NPi in nonsurvivors than survivors during the first 72 h^10^.

In patients following general anesthesia, pupillometric measurement parameters obtained by infrared pupillometry had an excellent performance predicting delirium at post-anesthesia care unit and were superior to other nonpupillary predictors^26^. Therefore, quantitative NPi can be suggested as an early prognostic predictor after cardiopulmonary compromise or anesthesia. Moreover, the pupillary light reflex was not abolished by adrenaline administration^27^, and the return of a normal pupillary reflex predicted the return of intact neurologic function in survivors of cardiopulmonary resuscitation^28^.

However, there were few studies evaluating intraoperative NPi and its relationship with postoperative delirium and clinical outcomes after cardiac surgery. We demonstrated the promising role of intraoperative NPi measurements to predict postoperative delirium following cardiac surgery with or without using CPB in this observational study.

Previously, most studies demonstrated that deeper anesthesia (lower BIS) has been associated with the development of postoperative delirium^29^. Similarly, we observed a moderate correlation of the lowest BIS values and the worst NPi during surgery in our cohort (see Supplementary Fig. S2 online). However, there were no differences in BIS values between patients who developed postoperative delirium and those did not (Table 2). Cerebral oxygen desaturation was not consistently correlated with postoperative delirium or cognitive dysfunction^29^, which was consistent with our results. In our cohort, patients with postoperative delirium had lower baseline and intraoperative cerebral oxygen saturation values, but the correlation between the lowest cerebral oxygen saturation and the lowest NPi was weak (r = 0.179; see Supplementary Fig. S2 online).

We used propofol and remifentanil as main anesthetics during the study period. In comparison of volatile anesthetics (sevoflurane) and propofol, NPi was well maintained under propofol-based anesthesia compared to sevoflurane throughout the surgery in a small study of pediatric patients undergoing elective surgery^30^. In another small study comparing the effects of anesthetics on NPi, there was no significant change in NPi during surgery in propofol/remifentanil group, while NPi significantly reduced from baseline in sevoflurane, sevoflurane/remifentanil and desflurane/remifentanil groups^31^. Although there is still limited evidence on the association of various anesthetics and pupillometric light reactivity, anesthetics used in this study might have minimal influence on NPi measurement during the surgery.

We observed that preoperative use of calcium channel blockers had a protective effect with lower risk of postoperative delirium following cardiac surgery (adjusted OR 0.257; 95% CI 0.007–0.857, P = 0.027; Table 3). Among the baseline characteristics of included subjects, it was observed that half of the patients who did not develop postoperative delirium had taken calcium channel blockers preoperatively, whereas only 33% of patients with postoperative delirium had used preoperative calcium channel blockers (Table 1). In a previous electronic medical records network study, calcium channel blockers were associated with higher incidence of delirium than renin-angiotensin system agents, but lower incidence compared to beta-blockers^32^. However, in a matched cohort study, brain-penetrant calcium channel blockers reduced risk ratios of neuropsychiatric disorders, including delirium, reflecting their blockade of neuronal voltage-gated calcium channels^33^. We could not distinguish differential effects of each type of calcium channel blocking agents in our analyses due to the lack of data. Therefore, our findings are not conclusive in the current analysis and more studies may be required to investigate the effects of calcium channel blockers on postoperative delirium following surgery.

This study had several limitations. First, this was a single-center observational study with limited number of subjects. Due to the small number of patients and delirium occurrence, the results from the multivariable logistic regression may have a model over-fitting problem. Moreover, due to the lack of pre-defined power calculation, this study has a low power. Further prospective large-scale studies may provide confirmatory implications on the prognostic value of intraoperative NPi and postoperative outcomes after cardiac surgery. Second, heterogenous cardiac disease pathology and underlying etiology have not been incorporated in this analysis. Further investigations are needed to evaluate the correlation of disease-specific clinical presentation and neurologic examination including pupillometry. Third, we did not evaluate the inflammatory markers, such as TNF-α or interleukin-6 in this study. However, postoperative C-reactive protein levels were comparable between the groups in our cohort. Fourth, the pupillometric findings were obtained during the use of opioids (remifentanil), which may impact the pupillary response^34^. In a small volunteer study, pupillary light reflex remained quantifiable under high-dose remifentanil administration inducing respiratory depression, but the amplitude of reflex was linearly affected^35^. However, in this study we used remifentanil in all subjects and evaluated the relationship of NPi and postoperative delirium as the primary study endpoint. Moreover, as above-mentioned, propofol/remifentanil anesthetic technique might have minimally influenced the NPi measurements during the study period. Lastly, we found 4/123 (3.3%) patients developed postoperative stroke but did not find any correlation between the laterality of the worst NPi and the laterality of the stroke lesion. Further well-designed study with sufficient number of patients may be required to assess any relationship between NPi and stroke lesion.

This is the first study that evaluated the association of intraoperative pupillometric measurements and postoperative delirium after cardiac surgery. Although the sample size is small and the statistical analyses are simple, the findings of this research would provide scientific base to conduct further research in this field.

In conclusion, abnormal intraoperative pupillary reactivity using automated infrared pupillometry was a strong independent predictor of postoperative delirium in patients undergoing cardiac surgery, irrespective of baseline patient characteristics and operative variables. Intraoperative application of pupillometer may have prognostic value for development of postoperative delirium, thereby enabling close surveillance and early intervention in high-risk patients. The findings of this study provide unique insights for predicting postoperative delirium following cardiac surgery and suggest a potential clinical applicability of intraoperative quantitative pupillometry in patients susceptible to develop delirium.

### Supplementary Information

Supplementary Table S1.

Supplementary Table S2.

Supplementary Table S3.

Supplementary Table S4.

Supplementary Table S5.

Supplementary Table S6.

Supplementary Table S7.

Supplementary Figure S1.

Supplementary Figure S2.

Supplementary Legends.

## Data Availability

The datasets generated and/or analyzed during this study are available from the corresponding author on reasonable request.
