# Supplementary material for: Intraoperative neurological pupil index and postoperative delirium and neurologic adverse events after cardiac surgery: an observational study
Source: Sci Rep. 2023 Aug 24;13:13838. doi: 10.1038/s41598-023-41151-z (PMC10449781; doi:10.1038/s41598-023-41151-z)
Supplement: Supplementary file 1 — Supplementary Table S1. [file 41598_2023_41151_MOESM1_ESM.docx]

**Supplementary Table S1.** Incidences of postoperative outcomes after cardiac surgery in patients with intraoperative worst NPi ≥ 3.0 or < 3.0.

|  | Total (n=123) | NPi ≥ 3.0 (n=92) | NPi < 3.0 (n=31) | P value |
| --- | --- | --- | --- | --- |
| Delirium | 24 (19.5%) | 14 (15.2%) | 10 (32.3%) | 0.038 |
| Stroke | 4 (3.3%) | 2 (2.2%) | 2 (6.5%) | 0.263 |
| Acute kidney injury | 43 (35.0%) | 30 (32.6%) | 13 (41.9%) | 0.346 |
| Newly initiated continuous renal replacement therapy | 11 (8.9%) | 6 (6.5%) | 5 (16.1%) | 0.142 |
| Mechanical circulatory support device | 6 (4.9%) | 3 (3.3%) | 3 (9.7%) | 0.335 |
| Extracorporeal membrane oxygenation | 4 (3.3%) | 2 (2.2%) | 2 (6.5%) | 0.263 |
| Intra-arterial balloon pump | 5 (4.1%) | 2 (2.2%) | 3 (9.7%) | 0.101 |
| In-hospital mortality | 3 (2.4%) | 1 (1.1%) | 2 (6.5%) | 0.156 |
| Cause of the death |  |  |  |  |
| Sepsis | 2 (1.6%) | 1 (1.1%) | 1 (3.2%) | 0.442 |
| Multi-organ failure | 1 (0.8%) | 0 (0.0%) | 1 (3.2%) | 0.252 |

Values are number (proportions). NPi, neurological pupil index.
