# Supplementary material for: Intraoperative neurological pupil index and postoperative delirium and neurologic adverse events after cardiac surgery: an observational study
Source: Sci Rep. 2023 Aug 24;13:13838. doi: 10.1038/s41598-023-41151-z (PMC10449781; doi:10.1038/s41598-023-41151-z)
Supplement: Supplementary file 2 — Supplementary Table S2. [file 41598_2023_41151_MOESM2_ESM.docx]

**Supplementary Table S2.** Comparison of characteristics of patients according to the worst intraoperative neurological pupil index during surgery.

|  | Worst NPi > 0  (n=99) | Worst NPi = 0  (n=24) | P value |
| --- | --- | --- | --- |
| Age, years | 65 (range, 29–87) | 60 (range, 31–80) | 0.189 |
| Male | 57 (57.6%) | 17 (70.8%) | 0.234 |
| STS-PROM, % | 1.38 (0.80–2.82) | 1.01 (0.57–2.50) | 0.378 |
| Preoperative LV EF, % | 60 (55–63) | 57 (55–63) | 0.666 |
| Baseline hematocrit, % | 38.2 (5.7) | 40.5 (5.5) | 0.081 |
| Type of surgery |  |  | 0.001 |
| CABG | 30 (30.3%) | 4 (16.7%) |  |
| Valve surgery | 51 (51.5%) | 8 (33.3%) |  |
| Aorta surgery | 2 (2.0%) | 5 (20.8%) |  |
| Aortic arch surgery | 0 (0.0%) | 4 (16.7%) |  |
| Combined surgery* | 8 (8.1%) | 5 (20.8%) |  |
| Other cardiac surgery^†^ | 8 (8.1%) | 2 (8.3%) |  |
| Redo surgery | 11 (11.1%) | 2 (8.3%) | 0.691 |
| Postoperative ICU stay, h | 50 (39–114) | 73 (27–108) | 0.861 |
| Postoperative hospital stay, day | 16 (12–23) | 16 (12–26) | 0.977 |

Values are number (proportions), median (interquartile range), or mean (standard deviation). Age was presented as median (range). CABG, coronary artery bypass graft; EF, ejection fraction; ICU, intensive care unit; LV, left ventricle; STS-PROM, the Society of Thoracic Surgeons Predicted Risk of Mortality.

* Combined surgery included concomitant valve, aorta, and/or coronary artery bypass graft surgery.

† Other cardiac surgery included repair of atrial septal defect, excision of intracardiac mass, myectomy, and endoventricular circular patch plasty.
