# Supplementary material for: Intraoperative neurological pupil index and postoperative delirium and neurologic adverse events after cardiac surgery: an observational study
Source: Sci Rep. 2023 Aug 24;13:13838. doi: 10.1038/s41598-023-41151-z (PMC10449781; doi:10.1038/s41598-023-41151-z)
Supplement: Supplementary file 3 — Supplementary Table S3. [file 41598_2023_41151_MOESM3_ESM.docx]

**Supplementary Table S3.** Perioperative variables in patients according to the worst intraoperative neurological pupil index during surgery.

|  | Worst NPi > 0  (n=99) | Worst NPi = 0  (n=24) | P value |
| --- | --- | --- | --- |
| Neurological pupil index |  |  |  |
| Baseline | 4.3 (4.1–4.6) | 4.5 (4.0–4.7) | 0.248 |
| After anesthesia induction | 3.7 (3.5–4.0) | 3.6 (3.2–3.9) | 0.054 |
| At initiation of CPB or coronary artery bypass | 4.1 (3.7­–4.3) | 3.8 (3.3–4.1) | 0.007 |
| At 30 min of CPB or coronary artery bypass | 3.8 (3.5–4.1) | 3.4 (2.8–3.6) | <0.001 |
| At 60 min of CPB or coronary artery bypass | 3.7 (3.5–4.0) | 2.8 (0.0–3.3) | <0.001 |
| At protamine infusion | 4.0 (3.7–4.2) | 3.7 (2.9–4.4) | 0.105 |
| End of surgery | 3.9 (3.7–4.3) | 3.7 (0.8–4.2) | 0.022 |
| Incidence of neurological pupil index of 0 |  |  |  |
| Baseline | 0 | 0 |  |
| After anesthesia induction | 0 | 4 (16.7%) |  |
| At initiation of CPB or coronary artery bypass | 0 | 2 (8.3%) |  |
| At 30 min of CPB or coronary artery bypass | 0 | 3 (12.5%) |  |
| At 60 min of CPB or coronary artery bypass | 0 | 7 (29.2%) |  |
| At protamine infusion | 0 | 5 (20.8%) |  |
| End of surgery | 0 | 6 (25.0%) |  |
| Bispectral index |  |  |  |
| Baseline | 97 (94–98) | 95 (89–97) | 0.040 |
| At initiation of CPB or coronary artery bypass | 41 (35–45) | 38 (23–41) | 0.007 |
| At protamine infusion | 44 (40–50) | 39 (32–45) | 0.010 |
| End of surgery | 45 (41–49) | 41 (38–46) | 0.019 |
| Lowest bispectral index | 34 (25–39) | 19 (0–33) | <0.001 |
| Cerebral oximeter, % |  |  |  |
| Right |  |  |  |
| Baseline | 61 (55–67) | 59 (51–71) | 0.625 |
| At initiation of CPB or coronary artery bypass | 62 (54–68) | 60 (46–71) | 0.829 |
| At protamine infusion | 64 (58–72) | 66 (59–70) | 0.659 |
| End of surgery | 61 (56–67) | 63 (53–67) | 0.864 |
| Left |  |  |  |
| Baseline | 62 (55–66) | 58 (52–71) | 0.764 |
| At initiation of CPB or coronary artery bypass | 63 (55–68) | 62 (45–69) | 0.469 |
| At protamine infusion | 65 (59–72) | 65 (57–72) | 0.821 |
| End of surgery | 61 (56–68) | 61 (55–69) | 0.693 |
| Duration of operation, min | 289 (79) | 355 (100) | 0.001 |
| Lowest core body temperature, °C | 30.4 (28.7–34.4) | 27.2 (24.7–30.9) | 0.001 |

Values are median (interquartile range) or mean (standard deviation). CPB, cardiopulmonary bypass; NPi, neurological pupil index.
