# Supplementary material for: Intraoperative neurological pupil index and postoperative delirium and neurologic adverse events after cardiac surgery: an observational study
Source: Sci Rep. 2023 Aug 24;13:13838. doi: 10.1038/s41598-023-41151-z (PMC10449781; doi:10.1038/s41598-023-41151-z)
Supplement: Supplementary file 4 — Supplementary Table S4. [file 41598_2023_41151_MOESM4_ESM.docx]

**Supplementary Table S4**. Multivariable logistic regression analysis for postoperative delirium in patients undergoing cardiac surgery.

|  | Unadjusted model | |  | Adjusted model | |
| --- | --- | --- | --- | --- | --- |
|  | OR (95% CI) | P value |  | OR (95% CI) | P value |
| Intraoperative pupillometry |  |  |  |  |  |
| Worst NPi (as continuous value) | 0.723 (0.542–0.963) | 0.026 |  | 0.621 (0.438–0.881) | 0.008 |
| Baseline characteristics |  |  |  |  |  |
| Age | 1.069 (1.016–1.126) | 0.011 |  |  |  |
| Male | 1.358 (0.553–3.339) | 0.505 |  |  |  |
| Body mass index | 0.976 (0.859–1.110) | 0.716 |  |  |  |
| Hematocrit | 0.908 (0.836–0.987) | 0.024 |  |  |  |
| STS-PROM | 1.410 (1.163–1.710) | <0.001 |  | 1.500 (1.184–1.901) | 0.001 |
| LV EF | 0.960 (0.922–1.001) | 0.053 |  |  |  |
| Comorbidity |  |  |  |  |  |
| Hypertension | 1.206 (0.493–2.950) | 0.682 |  |  |  |
| Diabetes mellitus | 1.429 (0.573–3.559) | 0.444 |  |  |  |
| Coronary artery disease | 2.259 (0.915–5.578) | 0.077 |  | 4.240 (1.375–13.079) | 0.012 |
| Previous MI or angina | 1.361 (0.502–3.684) | 0.545 |  |  |  |
| Chronic kidney disease | 3.625 (1.279–10.270) | 0.015 |  |  |  |
| Preoperative atrial fibrillation | 3.379 (1.347–8.477) | 0.009 |  |  |  |
| Previous stroke or TIA | 2.275 (0.624–8.299) | 0.213 |  |  |  |
| Chronic obstructive pulmonary disease | 2.214 (0.512–9.578) | 0.287 |  |  |  |
| Preoperative medication |  |  |  |  |  |
| ACEi or ARB | 0.664 (0.266–1.659) | 0.381 |  |  |  |
| Beta blocker | 0.800 (0.328–1.954) | 0.624 |  |  |  |
| Calcium channel blocker | 0.471 (0.185–1.200) | 0.114 |  |  |  |
| Diuretics | 2.500 (0.980–6.379) | 0.055 |  |  |  |
| Statin | 0.705 (0.287–1.735) | 0.447 |  |  |  |
| Benzodiazepine | 3.760 (0.927–15.256) | 0.064 |  |  |  |
| Intraoperative variables |  |  |  |  |  |
| Duration of operation | 1.006 (1.001–1.011) | 0.024 |  |  |  |
| Use of cardiopulmonary bypass | 0.960 (0.342–2.694) | 0.938 |  |  |  |
| Type of surgery |  | 0.895 |  |  |  |
| CABG | Ref |  |  |  |  |
| Valve surgery | 0.820 (0.306–2.274) | 0.699 |  |  |  |
| Aorta surgery | 0.208 (0.002–2.040) | 0.330 |  |  |  |
| Combined surgery* | 0.678 (0.114–2.971) | 0.638 |  |  |  |
| Other cardiac surgery^†^ | 0.917 (0.151–4.225) | 0.919 |  |  |  |
| Redo surgery | 2.993 (0.882–10.158) | 0.079 |  |  |  |
| Lowest core body temperature | 1.035 (0.913–1.173) | 0.588 |  |  |  |
| Lowest bispectral index | 0.993 (0.961–1.027) | 0.689 |  |  |  |
| Moderate desaturation of cerebral oximeter | 1.750 (0.662–4.626) | 0.259 |  |  |  |
| Severe desaturation of cerebral oximeter | 3.393 (0.706–16.305) | 0.127 |  |  |  |
| Total amount of infused remifentanil | 1.000 (1.000–1.000) | 0.807 |  |  |  |
| Intraoperative transfusion | 2.824 (1.034–7.710) | 0.043 |  |  |  |
| Intraoperative use of inotropic or vasoactive agent |  |  |  |  |  |
| Epinephrine | 2.000 (0.560–7.147) | 0.286 |  |  |  |
| Norepinephrine | 1.429 (0.442–4.619) | 0.551 |  |  |  |
| Dobutamine | 1.500 (0.544–4.135) | 0.433 |  |  |  |
| Nitroglycerin | 1.506 (0.603–3.764) | 0.381 |  |  |  |
| Postoperative use of benzodiazepine | 6.382 (1.909–21.334) | 0.003 |  | 5.370 (1.290–22.350) | 0.021 |

ACEi, angiotensin converting enzyme inhibitor; ARB, angiotensin; CI, confidence interval; CABG, coronary artery bypass graft; EF, ejection fraction; LV, left ventricle; MI, myocardial infarction; NPi, neurological pupil index; OR, odds ratio; STS-PROM, the Society of Thoracic Surgeons Predicted Risk of Mortality; TIA, transient ischemic attack.

* Combined surgery included concomitant valve, aorta, and/or coronary artery bypass graft surgery.

† Other cardiac surgery included repair of atrial septal defect, excision of intracardiac mass, myectomy, and endoventricular circular patch plasty.
