# Supplementary material for: Intraoperative neurological pupil index and postoperative delirium and neurologic adverse events after cardiac surgery: an observational study
Source: Sci Rep. 2023 Aug 24;13:13838. doi: 10.1038/s41598-023-41151-z (PMC10449781; doi:10.1038/s41598-023-41151-z)
Supplement: Supplementary file 5 — Supplementary Table S5. [file 41598_2023_41151_MOESM5_ESM.docx]

**Supplementary Table S5**. Multivariable logistic regression analysis for postoperative delirium in patients undergoing cardiac surgery excluding cases of worst intraoperative neurological pupil index of 0.

|  | Unadjusted model | |  | Adjusted model | |
| --- | --- | --- | --- | --- | --- |
|  | OR (95% CI) | P value |  | OR (95% CI) | P value |
| Intraoperative pupillometry |  |  |  |  |  |
| NPi ≥3.0 | Ref |  |  | Ref |  |
| NPi <3.0 | 4.179 (0.842–20.726) | 0.080 |  | 5.997 (0.869–41.374) | 0.069 |
| Baseline characteristics |  |  |  |  |  |
| Age | 1.076 (1.011–1.145) | 0.021 |  |  |  |
| Male | 1.255 (0.440–3.582) | 0.671 |  |  |  |
| Body mass index | 0.990 (0.857–1.145) | 0.896 |  |  |  |
| Hematocrit | 0.915 (0.830–1.010) | 0.077 |  |  |  |
| STS-PROM | 1.400 (1.135–1.727) | 0.002 |  | 1.479 (1.152–1.899) | 0.002 |
| LV EF | 0.959 (0.916–1.005) | 0.079 |  |  |  |
| Comorbidity |  |  |  |  |  |
| Hypertension | 2.021 (0.683–5.982) | 0.204 |  |  |  |
| Diabetes mellitus | 1.213 (0.418–3.521) | 0.722 |  |  |  |
| Coronary artery disease | 3.016 (1.014–8.973) | 0.047 |  | 4.931 (1.335–18.216) | 0.017 |
| Previous MI or angina | 2.033 (0.687–6.022) | 0.200 |  |  |  |
| Chronic kidney disease | 3.000 (0.872–10.318) | 0.081 |  |  |  |
| Preoperative atrial fibrillation | 4.180 (1.391–12.560) | 0.011 |  |  |  |
| Previous stroke or TIA | 0.792 (0.089–7.035) | 0.834 |  |  |  |
| Chronic obstructive pulmonary disease | 0.963 (0.105–8.805) | 0.973 |  |  |  |
| Preoperative medication |  |  |  |  |  |
| ACEi or ARB | 0.811 (0.281–2.337) | 0.697 |  |  |  |
| Beta blocker | 1.020 (0.358–2.905) | 0.970 |  |  |  |
| Calcium channel blocker | 0.360 (0.116–1.114) | 0.076 |  |  |  |
| Diuretics | 2.919 (0.943–9.039) | 0.063 |  |  |  |
| Statin | 0.824 (0.284–2.392) | 0.722 |  |  |  |
| Benzodiazepine | 4.179 (0.842–20.726) | 0.080 |  |  |  |
| Intraoperative variables |  |  |  |  |  |
| Duration of operation | 1.007 (1.000–1.013) | 0.051 |  |  |  |
| Use of cardiopulmonary bypass | 1.192 (0.351–4.046) | 0.779 |  |  |  |
| Type of surgery |  | 0.947 |  |  |  |
| CABG | Ref |  |  |  |  |
| Valve surgery | 0.737 (0.237–2.372) | 0.602 |  |  |  |
| Aorta surgery | 0.754 (0.005–10.919) | 0.885 |  |  |  |
| Combined surgery* | 1.450 (0.225–7.524) | 0.683 |  |  |  |
| Other cardiac surgery^†^ | 0.754 (0.070–4.518) | 0.788 |  |  |  |
| Redo surgery | 3.297 (0.844–12.874) | 0.086 |  |  |  |
| Lowest core body temperature | 1.015 (0.858–1.201) | 0.861 |  |  |  |
| Lowest bispectral index | 0.980 (0.930–1.032) | 0.441 |  |  |  |
| Moderate desaturation of cerebral oximeter | 0.884 (0.227–3.448) | 0.859 |  |  |  |
| Severe desaturation of cerebral oximeter | 0.498 (0.004–5.017) | 0.680 |  |  |  |
| Total amount of infused remifentanil | 1.000 (1.000–1.000) | 0.796 |  |  |  |
| Intraoperative transfusion | 2.646 (0.855–8.189) | 0.091 |  |  |  |
| Intraoperative use of inotropic or vasoactive agent |  |  |  |  |  |
| Epinephrine | 1.429 (0.270–7.561) | 0.675 |  |  |  |
| Norepinephrine | 1.312 (0.340–5.074) | 0.693 |  |  |  |
| Dobutamine | 1.244 (0.398–3.886) | 0.707 |  |  |  |
| Nitroglycerin | 2.073 (0.670–6.416) | 0.206 |  |  |  |
| Postoperative use of benzodiazepine | 3.897 (0.966–15.730) | 0.056 |  |  |  |

ACEi, angiotensin converting enzyme inhibitor; ARB, angiotensin; CI, confidence interval; CABG, coronary artery bypass graft; EF, ejection fraction; LV, left ventricle; MI, myocardial infarction; NPi, neurological pupil index; OR, odds ratio; STS-PROM, the Society of Thoracic Surgeons Predicted Risk of Mortality; TIA, transient ischemic attack.

* Combined surgery included concomitant valve, aorta, and/or coronary artery bypass graft surgery.

† Other cardiac surgery included repair of atrial septal defect, excision of intracardiac mass, myectomy, and endoventricular circular patch plasty.
