# Supplementary material for: Intraoperative neurological pupil index and postoperative delirium and neurologic adverse events after cardiac surgery: an observational study
Source: Sci Rep. 2023 Aug 24;13:13838. doi: 10.1038/s41598-023-41151-z (PMC10449781; doi:10.1038/s41598-023-41151-z)
Supplement: Supplementary file 6 — Supplementary Table S6. [file 41598_2023_41151_MOESM6_ESM.docx]

**Supplementary Table S6**. Multivariable logistic regression analysis for postoperative delirium in patients undergoing cardiac surgery using cardiopulmonary bypass.

|  | Unadjusted model | |  | Adjusted model | |
| --- | --- | --- | --- | --- | --- |
|  | OR (95% CI) | P value |  | OR (95% CI) | P value |
| Intraoperative pupillometry |  |  |  |  |  |
| NPi ≥ 3.0 | Ref |  |  | Ref |  |
| NPi < 3.0 | 1.474 (0.486–4.470) | 0.493 |  | 2.036 (0.590–7.021) | 0.260 |
| Baseline characteristics |  |  |  |  |  |
| Age | 1.073 (1.013–1.136) | 0.017 |  |  |  |
| Male | 0.875 (0.313–2.449) | 0.799 |  |  |  |
| Body mass index | 0.965 (0.837–1.112) | 0.678 |  |  |  |
| Hematocrit | 0.897 (0.816–0.986) | 0.024 |  |  |  |
| STS-PROM | 1.384 (1.128–1.698) | 0.002 |  | 1.478 (1.162–1.879) | 0.001 |
| LV EF | 0.972 (0.923–1.023) | 0.273 |  |  |  |
| Comorbidity |  |  |  |  |  |
| Hypertension | 1.344 (0.479–3.768) | 0.574 |  |  |  |
| Diabetes mellitus | 0.989 (0.314–3.117) | 0.985 |  |  |  |
| Coronary artery disease | 3.702 (1.180–11.616) | 0.025 |  |  |  |
| Previous MI or angina | 3.286 (0.819–13.185) | 0.093 |  |  |  |
| Chronic kidney disease | 3.221 (0.909–11.418) | 0.070 |  |  |  |
| Preoperative atrial fibrillation | 3.172 (1.072–9.385) | 0.037 |  |  |  |
| Previous stroke or TIA | 2.300 (0.516–10.248) | 0.275 |  |  |  |
| Chronic obstructive pulmonary disease | 3.000 (0.463–19.453) | 0.249 |  |  |  |
| Preoperative medication |  |  |  |  |  |
| ACEi or ARB | 0.571 (0.194–1.682) | 0.310 |  |  |  |
| Beta blocker | 0.667 (0.237–1.873) | 0.442 |  |  |  |
| Calcium channel blocker | 0.255 (0.068–0.954) | 0.042 |  | 0.182 (0.040–0.817) | 0.026 |
| Diuretics | 2.275 (0.737–7.020) | 0.153 |  |  |  |
| Statin | 0.738 (0.263–2.077) | 0.566 |  |  |  |
| Benzodiazepine | 2.800 (0.603–13.011) | 0.189 |  |  |  |
| Intraoperative variables |  |  |  |  |  |
| Duration of operation | 1.005 (1.000–1.011) | 0.047 |  |  |  |
| Type of surgery |  | 0.623 |  |  |  |
| CABG | Ref |  |  |  |  |
| Valve surgery | 0.263 (0.037–1.860) | 0.204 |  |  |  |
| Aorta surgery | 0.067 (0.001–1.183) | 0.144 |  |  |  |
| Combined surgery* | 0.217 (0.020–2.028) | 0.216 |  |  |  |
| Other cardiac surgery^†^ | 0.294 (0.027–2.823) | 0.329 |  |  |  |
| Redo surgery | 3.221 (0.909–11.418) | 0.070 |  |  |  |
| Lowest core body temperature | 1.099 (0.897–1.347) | 0.360 |  |  |  |
| Lowest bispectral index | 0.994 (0.957–1.032) | 0.741 |  |  |  |
| Moderate desaturation of cerebral oximeter | 1.312 (0.410–4.205) | 0.647 |  |  |  |
| Severe desaturation of cerebral oximeter | 2.219 (0.373–13.182) | 0.381 |  |  |  |
| Total amount of infused remifentanil | 1.000 (1.000–1.000) | 0.596 |  |  |  |
| Intraoperative transfusion | 2.054 (0.698–6.045) | 0.191 |  |  |  |
| Intraoperative use of inotropic or vasoactive agent |  |  |  |  |  |
| Epinephrine | 2.095 (0.565–7.776) | 0.269 |  |  |  |
| Norepinephrine | 1.026 (0.298–3.530) | 0.968 |  |  |  |
| Dobutamine | 0.952 (0.238–3.804) | 0.945 |  |  |  |
| Nitroglycerin | 2.230 (0.778–6.394) | 0.135 |  |  |  |
| Postoperative use of benzodiazepine | 5.750 (1.588–20.825) | 0.008 |  |  |  |

ACEi, angiotensin converting enzyme inhibitor; ARB, angiotensin; CI, confidence interval; CABG, coronary artery bypass graft; EF, ejection fraction; LV, left ventricle; MI, myocardial infarction; NPi, neurological pupil index; OR, odds ratio; STS-PROM, the Society of Thoracic Surgeons Predicted Risk of Mortality; TIA, transient ischemic attack.

* Combined surgery included concomitant valve, aorta, and/or coronary artery bypass graft surgery.

† Other cardiac surgery included repair of atrial septal defect, excision of intracardiac mass, myectomy, and endoventricular circular patch plasty.
