# Supplementary material for: Intraoperative neurological pupil index and postoperative delirium and neurologic adverse events after cardiac surgery: an observational study
Source: Sci Rep. 2023 Aug 24;13:13838. doi: 10.1038/s41598-023-41151-z (PMC10449781; doi:10.1038/s41598-023-41151-z)
Supplement: Supplementary file 7 — Supplementary Table S7. [file 41598_2023_41151_MOESM7_ESM.docx]

**Supplementary Table S7**. Multivariable logistic regression analysis for postoperative delirium in patients undergoing cardiac valve surgery.

|  | Unadjusted model | |  | Adjusted model | |
| --- | --- | --- | --- | --- | --- |
|  | OR (95% CI) | P value |  | OR (95% CI) | P value |
| Intraoperative pupillometry |  |  |  |  |  |
| NPi ≥ 3.0 | Ref |  |  | Ref |  |
| NPi < 3.0 | 2.857 (0.674–12.108) | 0.154 |  | 4.357 (0.730–25.991) | 0.106 |
| Baseline characteristics |  |  |  |  |  |
| Age | 1.068 (0.996–1.144) | 0.063 |  |  |  |
| Male | 0.723 (0.198–2.642) | 0.623 |  |  |  |
| Body mass index | 0.948 (0.780–1.151) | 0.588 |  |  |  |
| Hematocrit | 0.873 (0.769–0.990) | 0.034 |  |  |  |
| STS-PROM | 1.468 (1.143–1.885) | 0.003 |  | 1.746 (1.202–2.536) | 0.003 |
| LV EF | 0.968 (0.886–1.057) | 0.468 |  |  |  |
| Comorbidity |  |  |  |  |  |
| Hypertension | 0.806 (0.212–3.066) | 0.751 |  |  |  |
| Diabetes mellitus | 0.872 (0.204–3.734) | 0.853 |  |  |  |
| Coronary artery disease | 5.375 (1.109–26.045) | 0.037 |  |  |  |
| Previous MI or angina | 7.500 (1.092–51.518) | 0.040 |  |  |  |
| Chronic kidney disease | 2.437 (0.589–10.094) | 0.219 |  |  |  |
| Preoperative atrial fibrillation | 4.792 (0.946–24.269) | 0.058 |  |  |  |
| Previous stroke or TIA | 1.367 (0.239–7.811) | 0.725 |  |  |  |
| Chronic obstructive pulmonary disease | 2.933 (0.432–19.937) | 0.271 |  |  |  |
| Preoperative medication |  |  |  |  |  |
| ACEi or ARB | 0.450 (0.108–1.878) | 0.273 |  |  |  |
| Beta blocker | 1.043 (0.294–3.708) | 0.948 |  |  |  |
| Calcium channel blocker | 0.123 (0.015–1.030) | 0.053 |  | 0.031 (0.002–0.593) | 0.021 |
| Diuretics | 2.581 (0.504–13.220) | 0.255 |  |  |  |
| Statin | 0.209 (0.041–1.057) | 0.058 |  |  |  |
| Benzodiazepine | 2.933 (0.432–19.937) | 0.271 |  |  |  |
| Intraoperative variables |  |  |  |  |  |
| Duration of operation | 1.011 (1.003–1.020) | 0.011 |  |  |  |
| Redo surgery | 4.082 (1.006–16.563) | 0.049 |  |  |  |
| Lowest core body temperature | 1.149 (0.801–1.649) | 0.450 |  |  |  |
| Lowest bispectral index | 1.001 (0.941–1.065) | 0.975 |  |  |  |
| Moderate desaturation of cerebral oximeter | 2.643 (0.690–10.129) | 0.156 |  |  |  |
| Severe desaturation of cerebral oximeter | 2.150 (0.344–13.424) | 0.413 |  |  |  |
| Total amount of infused remifentanil | 1.000 (1.000–1.001) | 0.739 |  |  |  |
| Intraoperative transfusion | 2.087 (0.552–7.887) | 0.278 |  |  |  |
| Intraoperative use of inotropic or vasoactive agent |  |  |  |  |  |
| Epinephrine | 1.367 (0.239–7.811) | 0.725 |  |  |  |
| Norepinephrine | 1.029 (0.238–4.437) | 0.970 |  |  |  |
| Dobutamine | 0.595 (0.100–3.526) | 0.568 |  |  |  |
| Nitroglycerin | 2.471 (0.676–8.999) | 0.170 |  |  |  |
| Postoperative use of benzodiazepine | 4.200 (0.922–19.140) | 0.064 |  |  |  |

ACEi, angiotensin converting enzyme inhibitor; ARB, angiotensin; CI, confidence interval; EF, ejection fraction; LV, left ventricle; MI, myocardial infarction; NPi, neurological pupil index; OR, odds ratio; STS-PROM, the Society of Thoracic Surgeons Predicted Risk of Mortality; TIA, transient ischemic attack.
