# Supplementary figures and images for: Intraoperative neurological pupil index and postoperative delirium and neurologic adverse events after cardiac surgery: an observational study
Source: Sci Rep. 2023 Aug 24;13:13838. doi: 10.1038/s41598-023-41151-z (PMC10449781; doi:10.1038/s41598-023-41151-z)

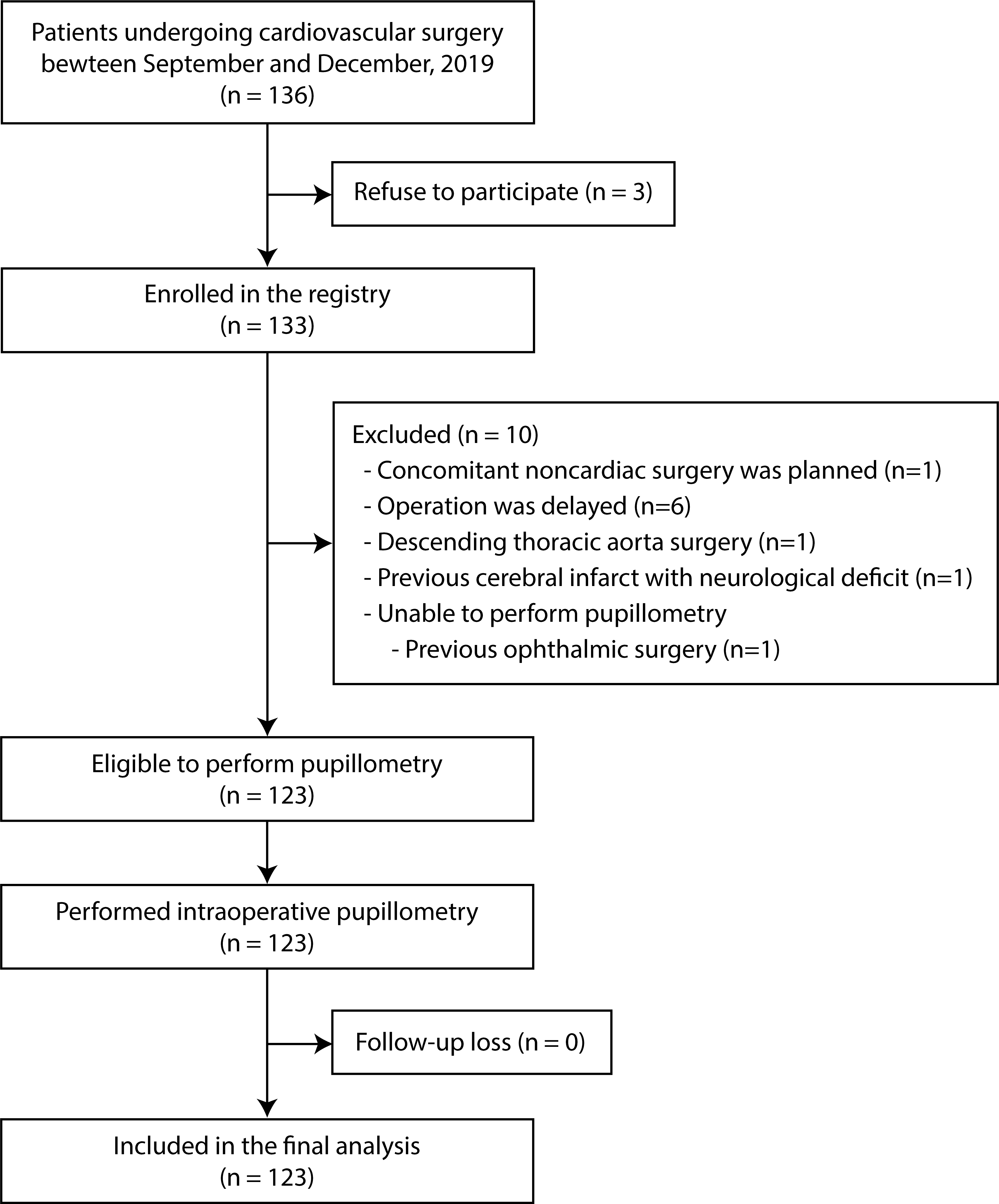

Supplement: Supplementary file 8 — Supplementary Figure S1. [file 41598_2023_41151_MOESM8_ESM.tif]

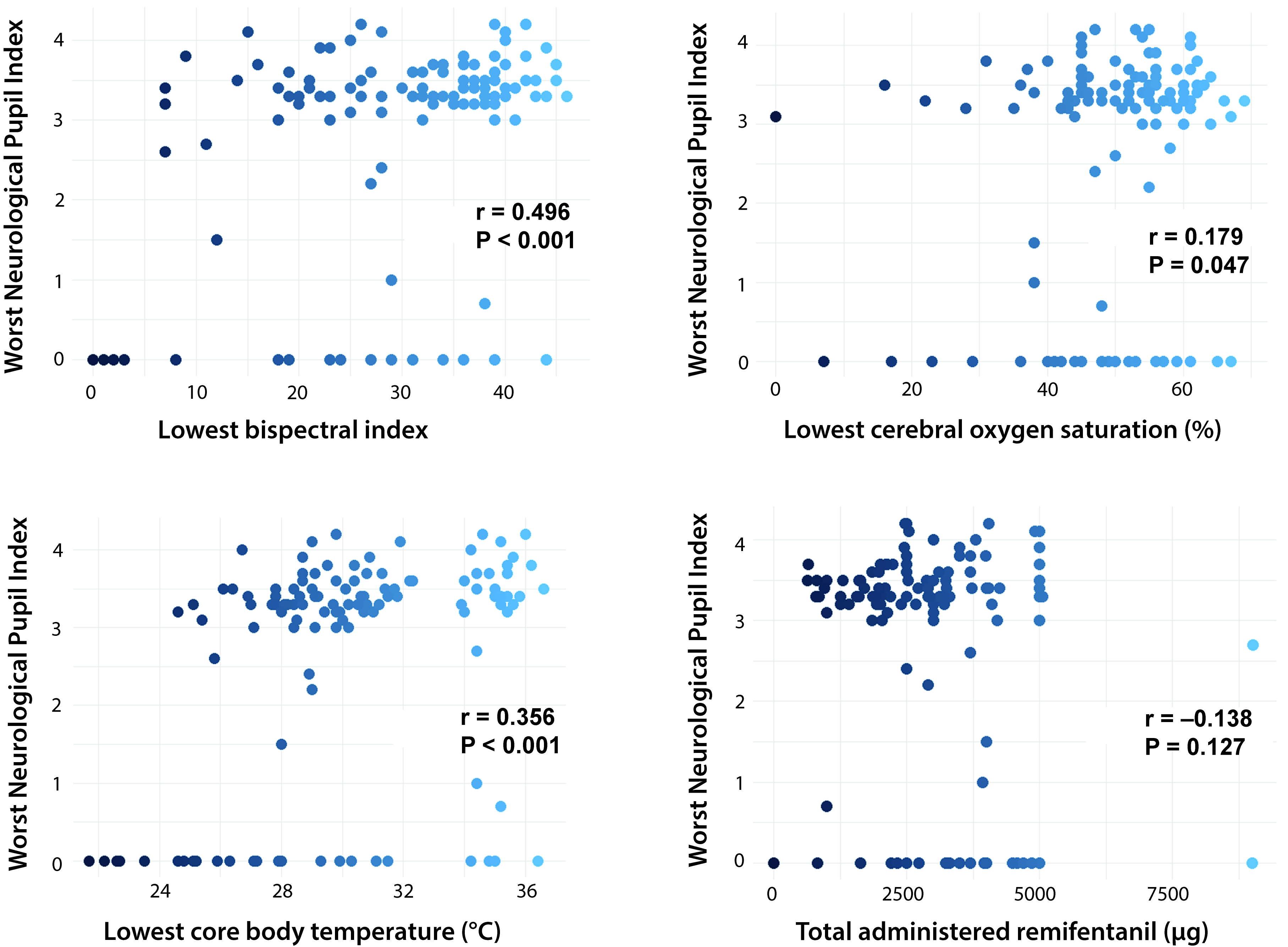

Supplement: Supplementary file 9 — Supplementary Figure S2. [file 41598_2023_41151_MOESM9_ESM.tif]
