# Supplementary material for: Intraoperative neurological pupil index and postoperative delirium and neurologic adverse events after cardiac surgery: an observational study
Source: Sci Rep. 2023 Aug 24;13:13838. doi: 10.1038/s41598-023-41151-z (PMC10449781; doi:10.1038/s41598-023-41151-z)
Supplement: Supplementary file 10 — Supplementary Legends. [file 41598_2023_41151_MOESM10_ESM.docx]

**Supplementary Figure Legends**

**Supplementary Figure S1**. STROBE diagram of study.

**Supplementary Figure S2**. Scatter plots for correlations between the worst neurological pupil index versus lowest bispectral index, lowest cerebral oxygen saturation, lowest core body temperature, and total administered remifentanil during cardiac surgery.
